# Phospholipase C-η2 interacts with nuclear and cytoplasmic LIMK-1 during retinoic acid-stimulated neurite growth

**DOI:** 10.1007/s00418-015-1390-7

**Published:** 2015-12-15

**Authors:** Mohammed Arastoo, Christian Hacker, Petra Popovics, John M. Lucocq, Alan J. Stewart

**Affiliations:** School of Medicine, Medical and Biological Sciences Building, North Haugh, University of St Andrews, St Andrews, Fife KY16 9TF UK; Bioimaging Centre, Geoffrey Pope Building, College of Life and Environmental Sciences, University of Exeter, Exeter, EX4 4QD UK; Veterans Affairs Medical Center, Miami, FL 33125 USA

**Keywords:** Calcium signaling, Cell differentiation, Electron microscopy, Neuritogenesis, Protein–protein interaction

## Abstract

Neurite growth is central to the formation and differentiation of functional neurons, and recently, an essential role for phospholipase C-η2 (PLCη2) in neuritogenesis was revealed. Here we investigate the function of PLCη2 in neuritogenesis using Neuro2A cells, which upon stimulation with retinoic acid differentiate and form neurites. We first investigated the role of the PLCη2 calcium-binding EF-hand domain, a domain that is known to be required for PLCη2 activation. To do this, we quantified neurite outgrowth in Neuro2A cells, stably overexpressing wild-type PLCη2 and D256A (EF-hand) and H460Q (active site) PLCη2 mutants. Retinoic acid-induced neuritogenesis was highly dependent on PLCη2 activity, with the H460Q mutant exhibiting a strong dominant-negative effect. Expression of the D256A mutant had little effect on neurite growth relative to the control, suggesting that calcium-directed activation of PLCη2 is not essential to this process. We next investigated which cellular compartments contain endogenous PLCη2 by comparing immunoelectron microscopy signals over control and knockdown cell lines. When signals were analyzed to reveal specific labeling for PLCη2, it was found to be localized predominantly over the nucleus and cytosol. Furthermore in these compartments (and also in growing neurites), a proximity ligand assay revealed that PLCη2 specifically interacts with LIMK-1 in Neuro2A cells. Taken together, these data emphasize the importance of the PLCη2 EF-hand domain and articulation of PLCη2 with LIMK-1 in regulating neuritogenesis.

## Introduction

Phospholipase C (PLC) enzymes are a well-characterized family of hydrolytic enzymes (E.C. 3.1.4.11). In mammals, PLC enzymes are responsible for cleaving the membrane phospholipid phosphatidylinositol 4,5-bisphosphate (PtdIns(4,5)P_2_), thereby generating the two essential second messengers inositol 1,4,5-trisphosphate (Ins(1,4,5)P_3_) and 1,2-diacylglycerol (DAG) following receptor activation. Consequently, Ins(1,4,5)P_3_ triggers calcium discharge from the endoplasmic reticulum and DAG together with calcium activates effector proteins, most notably protein kinase C (PKC; Berridge et al. 1983; Hokin and Hokin 1953; Streb et al. 1983). Six classes of PLCs have been identified based on their amino acid sequence, domain structure, and mechanism of activation. These include the β, γ, δ, ε, ζ and η classes (Suh et al. 2008). The most recently identified is the η class of which there are two isoforms, PLCη1 and PLCη2 (Hwang et al. 2005; Nakahara et al. 2005; Stewart et al. 2005; Zhou et al. 2005), encoded by the *PLCH1* and *PLCH2* genes, respectively. A number of spliceforms of both PLCη1 and PLCη2 exist, which vary in length at the C-terminal end (Hwang et al. 2005; Zhou et al. 2005). This region is rich in serine and proline residues, and it is thought that this part of the protein may facilitate protein–protein interactions (Suh et al. 2008). Both PLCη enzymes can be activated directly by mobilization of intracellular calcium (Kim et al. 2011; Popovics et al. 2011, 2014). We recently reported that mutation of a putative calcium-binding residue (D256A) within EF-loop 1 of the EF-hand domain of PLCη2 reduces the sensitivity to calcium by tenfold, indicating this domain to be responsible for calcium-induced activation (Popovics et al. 2014). In addition, it has also been shown that PLCη2, but not PLCη1, can be activated by Gβγ, which is released from trimeric G-protein complexes following G-protein-coupled receptor activation (Zhou et al. 2005, 2008).

The isozyme PLCη2 is expressed in neurons with highest expression in the olfactory bulb, cerebral cortex and pyramidal cells of the hippocampus (Nakahara et al. 2005). It is also expressed within the habenula, the retina (Kanemura et al. 2010), in the pituitary and neuroendocrine cells (Stewart et al. 2007) as well as in non-nervous tissue such as the intestine and pancreatic islets (Stewart et al. 2005). Its specific function(s) within neurons is still unclear, although considering its sensitivity toward calcium, it is thought that PLCη2 may act synergistically with other PLCs or calcium-activated processes (Popovics and Stewart 2012). PLCη2 is expressed in the brain of mice after birth and increases until 4 weeks of age. Cultured hippocampal pyramidal cells showed a high level of PLCη2, whereas astrocyte-enriched cultures did not show any expression (Nakahara et al. 2005), indicating a functional role in neuronal formation and physiology. In accordance, deletion of the chromosomal region 1p36.32 in which PLCη2 is located, leads to mental retardation (Fitzgibbon et al. 2008; Lo Vasco 2011). Previously, using a targeted knockdown approach, we found PLCη2 to be essential for retinoic acid-induced neurite growth in Neuro2A cells (Popovics et al. 2013). Despite this, the precise mechanism by which PLCη2 facilitates neurite growth in these cells is not known. In our previous study, through use of a bacterial two-hybrid assay we identified Lim-domain kinase-1 (LIMK-1) as a putative C-terminal interaction partner of PLCη2 (Popovics et al. 2013). LIMK-1 acts primarily downstream of Rho GTPases to phosphorylate and inactivate cofilin family proteins (cofilin1, cofilin2 and destrin), which promote actin depolymerization and the severing of actin filaments during neurite outgrowth (Arber et al. 1998; Yang et al. 1998; Endo et al. 2007). LIMK-1 has also been shown to be a substrate for calcium/calmodulin-dependent kinase-IV (CaMKIV) during neurite growth (Takemura et al. 2009). Although it was revealed that PLCη2 and LIMK-1 do indeed co-localize in Neuro2A cells (Popovics et al. 2013), it is not known whether these two proteins directly interact in the cell, or whether such interactions occur during neurite outgrowth.

To gain a better understanding of the role of PLCη2 in neuritogenesis, we examine the importance of PLCη2 activity and calcium-mediated activation of the enzyme for neurite outgrowth in Neuro2A cells stably overexpressing wild-type and mutant PLCη2 proteins. In addition, we examine the intracellular localization of PLCη2 in Neuro2A cells at the ultrastructural level and, through use of a proximity ligand assay, probe the interaction of PLCη2 and LIMK-1 in differentiating Neuro2A cells.

## Materials and methods

### Growth and retinoic acid-induced differentiation of Neuro2A cells

Neuro2A cells were obtained from the European Collection of Cell Cultures (Salisbury, UK). Eagle’s minimal essential medium (EMEM) supplemented with 10 % fetal bovine serum (FBS), 2 mM l-glutamine and 50 units/ml of penicillin/streptomycin (complete EMEM) was used to maintain Neuro2A cells and stable transfected clones. Neuro2A cells were differentiated as previously described by Zeng and Zhou (2008). Briefly, cells were plated at a low density (100 cells/mm^2^) in 6-well plates in complete EMEM which was changed to the differentiation medium the next day (EMEM with 2 % FBS, 2 mM l-glutamine and 20 µM retinoic acid). Medium was replaced every day for 4 days. Micrographs showing cells at 4-day differentiation were collected by a Zeiss Axiovert 40 CFL microscope with a 10× objective (Carl Zeiss Ltd., Cambridge, UK). Images were taken using an AxioCam ICc 1 digital camera (Carl Zeiss Ltd.). Neurite outgrowth was assessed by taking four micrographs by random selection from all experimental conditions (in each case, at least 220 cells were sampled per experiment), and experiments were repeated three times. All cells possessing at least one neurite with a length at least twice the cell body were considered differentiated. Results were expressed as a percentage of differentiated/total cell number.

### Generation of stable Neuro2A cell lines overexpressing wild-type and mutant PLCη2

An expression construct encoding residues 75-1238 of mouse PLCη2 (isoform a; NP_780765) in pcDNA3.1 (as used in Nakahara et al. 2005) was a gift from Prof. Kiyoko Fukami (Tokyo University of Pharmacy and Life Science, Japan). The pcDNA3.1-PLCη2 expression construct was used to synthesize D256A and H460Q mutants using the Quikchange Site-Directed Mutagenesis Kit (Stratagene, Amsterdam, The Netherlands). Neuro2A cells were stably transfected with empty pcDNA3.1 vector (control) and wild-type and mutant PLCη2 expressing plasmids to produce cell lines overexpressing each protein. In each case, 20 µg of DNA was transfected by electroporation using a Bio-Rad Gene Pulser (Hertfordshire, UK) at 230 V, 950 μF. Stably transfected cells were selected after 48 h by adding 500 µg/ml G418 (InvivoGen, San Diego, CA, USA) and single cell-derived clones were picked and cultured for further experiments.

### Measurement of PLCH2 mRNA levels in stable Neuro2A cell lines

The mRNA expression levels of *PLCH2* (OMIM *612836) forms in stable Neuro2A cell lines were measured using quantitative PCR. Briefly, mRNA was isolated using the Isolate RNA Mini Kit (Bioline, London, UK) according to the manufacturer’s instructions. DNA contamination was removed by DNAse digestion (RQ1 RNase-Free DNase, Promega, Southampton, UK). This was followed by a two-step reverse transcription reaction using 0.5 µg of mRNA. The mRNA was incubated with 100 pmol oligod T_18_ and 0.5 mM dNTP in DEPC-treated water at 65 °C for 5 min. RevertAid Premium Reverse Transcriptase (200 units), RiboLock RNase Inhibitor (20 units; Thermo Fisher Scientific, Surrey, UK) and 1× RT-buffer (Fermentas, St. Leon-Rot, Germany) were added. Mixtures were incubated for 30 min at 50 °C followed by 10 min at 60 °C. Reactions were terminated at 85 °C for 5 min. *PLCH2* and *RPLP0* mRNA expression levels were detected by RT-PCR using primers obtained from Eurofins Scientific (Luxembourg). The sequences of corresponding primers used were: *PLCH2*, 5′-GGCTACACTCTGACCTCCAAGATCC-3′ (forward) and 5′-GGAAGCATGGTGGCATCTTCGCTGC-3′ (reverse); *RPLP0*, 5′-GAGTGATGTGCAGCTGATAAAGACTGG-3′ (forward) and 5′-CTGCTCTGTGATGTCGAGCACTTCAG-3′ (reverse). The *RPLP0* primers were used previously as described (Popovics et al. 2013). The *PLCH2* primers were designed in house and checked for specificity using adequate positive and negative controls. PCR reactions contained 1× reaction buffer, 1.5 mM MgCl_2_, 0.2 mM dNTPs, 250 nM of each primer and 1.25 units of GoTaq polymerase (Promega). Reactions were cycled at 95 °C for 15 s, 60 °C for 30 s and 72 °C for 1 min. The expression level of PLCη2 was measured by qPCR relative to the level of the large ribosomal protein P0 (RPLP0) mRNA. Expression was detected by Brilliant III Ultra-Fast SYBR Green mix (Agilent Technologies, Cheshire, UK) using a 7300 Real-Time PCR system (Applied Biosystems, Massachusetts, USA). Expression levels were calculated by the ΔC_*t*_ method. At least four colonies were chosen from each stably transfected cell group, and qPCR analysis was performed simultaneously on their cDNA to evaluate the expression of inserted gene. Based on the gene expression, colonies that possessed comparable levels of *PLCH2* in each transfection group were chosen for differentiation studies.

### Immunogold labeling and electron microscopy (EM)

Samples were processed according to the Tokuyasu thawed frozen section method (Tokuyasu 1973). Briefly, Neuro2A cells stably expressing a PLCη2-targetted shRNA (PLCη2 KD) or non-target shRNA control (referred to as shRNAPLCη2-1 and shRNA control, respectively, in Popovics et al. 2014) were grown to full confluency in a T-75 flask before fixation with 4 % *p*-formaldehyde, 0.05 % glutaraldehyde, buffered with 0.2 M PIPES, pH 7.2, for 15 min at ambient temperature. Cells were then scraped, collected and centrifuged at 16,000×*g* for 15 min to form a pellet before cryoprotection in 2.3 M sucrose in PBS (overnight at 4 °C). Small blocks were prepared from the pellets and mounted onto specimen carriers before plunge-freezing in liquid N_2_. Sections (80 nm thick) were cut at −100 °C (Leica EM FC7 ultracryomicrotome; Vienna, Austria) and retrieved using droplets of 2.1 M sucrose:2 % (w/v) methyl cellulose (mixed 1:1) and mounted in the same solution on pioloform-coated EM copper grids (Agar Scientific, Stanstead, UK) and stored at 4 °C. Prior to immunogold labeling, grids were washed three times in ice-cold distilled water (5 min each) and once in PBS (5 min) at ambient temperature. Sections were then incubated in 0.5 % fish skin gelatin (Sigma-Aldrich) in PBS and labeled using a custom polyclonal rabbit antibody raised commercially against a short peptide of PLCη2 (60 min; SKVEEDVEAGEDSGVSRQN; EZBiolab, Westfield, IN, USA), followed by three washes in PBS (15 min total) and 10 nm protein A-gold (20 min; BBI Solutions, Dundee, UK). After washing in PBS and distilled water, the sections were contrasted using 2 % (w/v) methylcellulose/3 % (w/v) uranyl acetate (mixed 9:1), and after air-drying the sections were visualized using a JEOL 1200 EX transmission electron microscope operated at 80 kV and images observed and recorded using an Orius 200 digital camera (Gatan, Abingdon, UK). Sections from shRNAPLCη2-1 and shRNA control samples were always run in parallel using the same solutions, dilutions and under environmental conditions.

Cell structures were identified according to the following criteria: The nuclear envelope was a double membrane that separated the nucleus from the cytosol. Mitochondria were elongated or circular profiles with double membranes and at least one double-membraned crista profile. ER was defined as a double-membraned cisterna decorated by ribosomes. The plasma membrane was a single membrane layer at the cell periphery. The Golgi apparatus cisternal stack was identified as at least two closely stacked cisternal membranes each with an axial ratio of at least 3:1 (groups of vesiculotubular structures were considered as belonging to Golgi if they were closer than two vesicle widths from Golgi cisternal structures). MVBs were round structures containing at least one round structure within it. Isolated vesicles were small round structures (50–100 nm) within the cytosol.

To analyze specific labeling, the control and knockdown preparations were fixed, sectioned and labeled in parallel using identical solutions. For quantification, labeled sections were visualized at a nominal magnification of 5000× and analyzed in a series of scans placed systematic uniform random (SUR; Lucocq 2008, 2012), across the ribbon of sections. Gold particles were counted and assigned to different cellular compartments. To compare the intensity of labeling, membranes of the endoplasmic reticulum or nuclear envelope were used as a standard to which gold labeling of various organelles/compartments was related. These standard membranes were assessed during scanning by counting intersections of these membranes with the edge of a marker feature that was placed on the display screen. The density of gold particles was then expressed as a labeling index by dividing the number of gold particles by the membrane intersections. In the case of gold labeling situated over volume occupying compartments such as nucleoplasm and cytosol, the gold counts were related to the number of nuclear envelope and plasma membrane intersections, respectively.

### Proximity ligand assay of PLCη2-LIMK-1 interactions

Ligand proximity assays as described by Söderberg et al. (2006) were performed using the Duolink proximity ligand assay system (Sigma-Aldrich). Two sets of experiments were performed; the first with Neuro2A cells stably expressing PLCη2-targeted shRNA and the corresponding non-target shRNA-expressing control cells and the second, with untransfected Neuro2A cells grown for 4 days in the presence and absence of 20 μM retinoic acid. In all cases, cells were seeded at a low density (100 cells/mm^2^) and grown on coverslips overnight. The PLA assay was performed in accordance with the manufacturer’s instructions. For specific visualization of the PLCη2-LIMK-1 interaction in cells, a custom rabbit anti-PLCη2 antibody (as described above; 1:100 dilution) and a mouse polyclonal anti-LIMK-1 antibody (Abcam, Cambridge, UK; 1:100 dilution) were used. Samples were analyzed using the Leica TCS SP8 confocal microscope with a 63× objective (Leica Microsystems, Heidelberg, Germany).

## Results

### PLCη2 activity is important for neurite growth

**Fig. 1:**
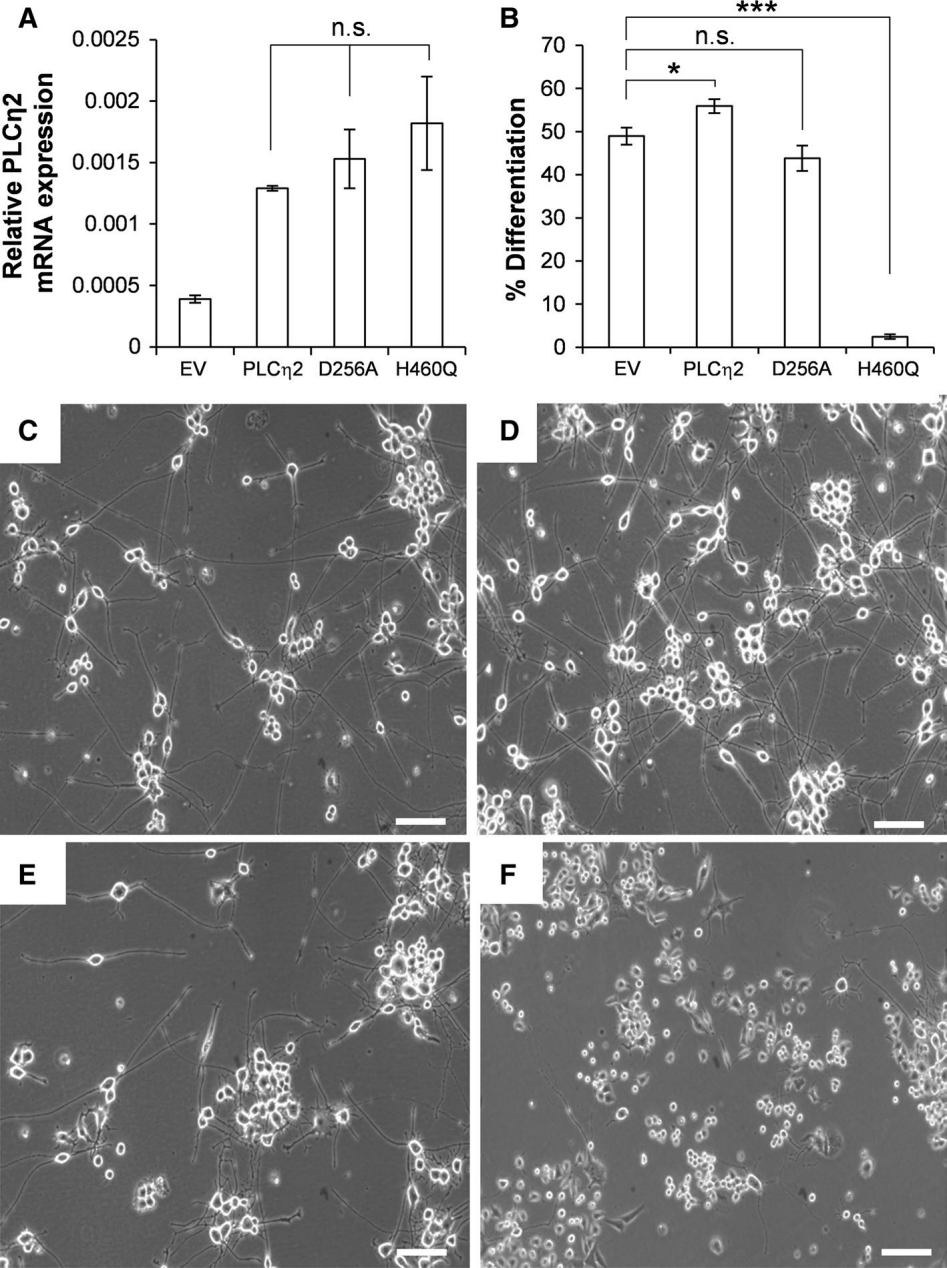
**a** *Graph* showing mRNA expression levels of PLCη2 relative to RPLP0 in stably transfected cell lines. *Error bars* represent S.E.M. The statistical significance was established by one-way ANOVA using Tukey’s honest significance test. Differences in PLCη2 mRNA expression (relative to RPLP0 mRNA expression) in cell lines stably expressing PLCη2 forms were nonsignificant as indicated by n.s; where *p* > 0.05. **b** *Graph* showing percentage of differentiation in different stable cell lines. *Error bars* represent S.E.M. The statistical significance (as determined by unpaired *t* test with applied Bonferroni’s correction) is indicated as ***p < 0.001 and *p < 0.05. **c**–**f** Representative *bright-field* images of stable cells expressing empty vector (EV), wild-type PLCη2 (PLCη2) and D256A and H460Q mutants, respectively, after 4 days of retinoic acid treatment. *Scale bars* correspond to 10 µm

A characteristic property of PLCη enzymes is their ability to be directly activated by calcium released from intracellular stores (Kim et al. 2011; Popovics et al. 2011). To assess the importance of PLCη2 activity in neurite outgrowth and to determine whether calcium-induced activation of PLCη2 plays a role in this process, Neuro2A cells stably overexpressing wild-type PLCη2 and mutant forms of the enzyme (D256A and H460Q) were created and analyzed together. Neuro2A cells stably transfected with empty vector only (EV) were used as a control. The D256A mutation has been demonstrated previously to abolish the ability of calcium to activate PLCη2 through perturbation of calcium binding at EF-loop 1 of the EF-hand domain (Popovics et al. 2014). His460 corresponds to a key active site residue that is highly conserved within PLC isozymes (Heinz et al. 1998), and mutation of the corresponding His residue in PLCδ1 (His356) has been shown to abolish the activity of this enzyme (Stallings et al. 2005). Quantitative PCR analysis revealed PLCη2 mRNA levels to be increased 3–4-fold in all cell lines stably expressing PLCη2 forms relative to control cells (Fig. 1a). The cell lines were treated with retinoic acid for 4 days, and the proportion of cells showing signs of differentiation was assessed (Fig. 1b–f). Following retinoic acid treatment, there was a slight but significant increase in the average percentage of differentiated cells observed in the wild-type PLCη2 stably transfected cells (55.9 ± 0.9 %), relative to controls cells (49.0 ± 1.1 %), *p* = 0.0285, *n* = 3. Conversely, cells stably transfected with the H460Q mutant exhibited a dramatic reduction in the proportion of differentiated cells (2.4 ± 0.3 %) relative to control cells after stimulation with retinoic acid, *p* = 0.0003, *n* = 3. A comparable degree of differentiation was observed in the cells stably transfected with the D256A mutant (44.9 ± 0.9 %) relative to control after the treatment, *p* = 0.9195, *n* = 3. Note that errors quoted above represent S.E.M from three separate experiments. To reduce the possibility of type 1 errors, *p* values were corrected by applying Bonferroni’s correction.

### Immunogold localization of PLCη2 in Neuro2A cells

**Fig. 2:**
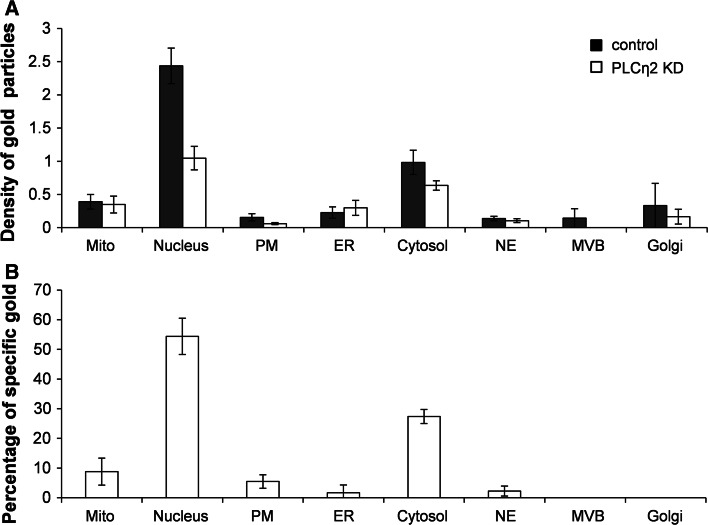
**a** *Graph* showing the labeling index for PLCη2 localization over cell compartments and organelles in non-target shRNA control cells (*black bars*) and PLCη2 KD cells (*white bars*). The index was calculated by dividing the number of counted gold particles counted over each organelle by the number of intercepts that a set reference point made with standard membrane features during scanning. *Error bars* represent S.E.M from three experiments. *Filled bars* control cells and *open bars* knockdown. **b** *Graph* showing the percentage of specific gold in each compartment/organelle as derived from Table 1. *Error bars* represent S.E.M from three experiments. *Mito* mitochondria, *PM* plasma membrane, *ER* endoplasmic reticulum, *NE* nuclear envelope, *MVB* multivesicular body, *Golgi* Golgi apparatus

Because of the compartmentalized nature of cells, determining a protein’s localization can provide a good indication of its functional role. Therefore, we examined the intracellular localization of endogenous PLCη2 in Neuro2A cells using quantitative immunoelectron microscopy (EM). We used two previously generated Neuro2A cell lines (Popovics et al. 2013): one stably expressing PLCη2-targeted shRNA plasmid (PLCη2 KD) and the other a non-target shRNA (control). As determined by Western blotting, the cell line expressing the PLCη2-targetted shRNA plasmid was shown to exhibit a 67 % reduction in PLCη2 expression at the protein level, and a ninefold reduction in retinoic acid-induced neuritogenesis after 4 days, relative to the control cells (Popovics et al. 2013). The signal intensities over a range of cellular compartments were compared as shown in Fig. 2a. The majority of organelles were found to exhibit a higher signal in the control compared with PLCη2 KD cells. The specific percentage of gold particles attributed to each organelle was then calculated in order to assess the degree of specific staining (Fig. 2b; Table 1). The majority of specific signal, corresponding to the presence of PLCη2, was found in the cell nucleus (54.4 ± 3.5 %) and to a lesser degree, the cytosol (27.4 ± 1.4 %) and mitochondria (8.8 ± 2.6 %), A smaller specific signal was observed at plasma membrane (5.5 ± 1.3 %), nuclear envelope (2.2 ± 1.0 %), endoplasmic reticulum (1.7 ± 1.5 %). No specific staining was observed over the Golgi apparatus or multivesicular bodies. Note that errors quoted above represent S.E.M from three separate experiments.

**Table 1:** Data used to calculate the specific percentage of gold particles in cellular compartments/organelles

	Specific density (SD)	Fraction specific (FS)	Total raw gold	Specific gold (SG)	% Specific
Mitochondria	0.16	0.48	25.33	9.59	8.82
Nucleus	1.74	0.72	102.00	71.60	54.38
PM	0.12	0.75	9.67	7.15	5.47
ER	0.04	0.21	19.67	1.23	1.71
Cytosol	0.56	0.57	62.67	35.77	27.38
NE	0.07	0.53	5.00	2.29	2.25
MVB	0.14	0.33	1.00	0.00	0.00
Golgi	0.22	0.22	0.33	0.00	0.00

Specific labeling density was calculated from the difference between control and knockdown. The number of specific gold particles attributed to each organelle was calculated as described previously (Lucocq and Gawden-Bone 2010). Briefly, specific density (SD) of gold particles was calculated by subtracting the average density of labeling for each organelle in PLCη2 KD Neuro2A cells from that of control cells. SD was then divided by the average density in control cells to determine the fraction specific (FS) gold particles. Next, specific gold (SG) was calculated by multiplying FS by the total number of raw gold particles counted in each organelle in control cells. This was then divided by the total SG across all compartments and multiplied by 100 to establish the percentage of specific gold in each organelle. Data are expressed as average values across three experiments

### PLCη2 interacts directly with LIMK-1

**Fig. 3:**
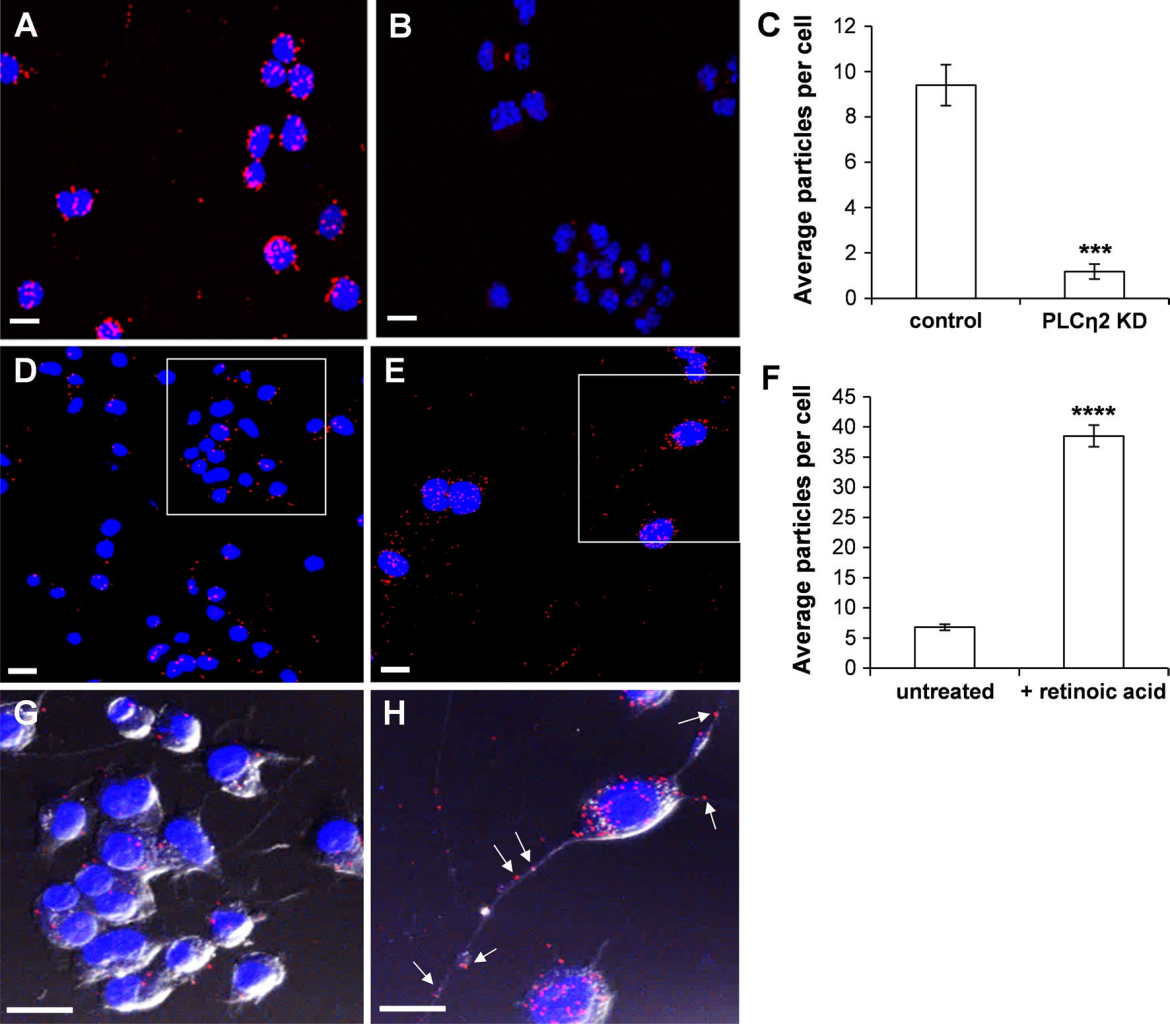
Proximity ligand assay allowing visualization and quantification of PLCη2-LIMK-1 interactions in Neuro2A cells. Fluorescent images in (**a**) and (**b**) are taken from control and PLCη2 KD Neuro2A cells, respectively. Fluorescent particles corresponding to PLCη2-LIMK-1 interactions appear in *red* and DAPI-staining of nuclei in *blue*. These micrographs are representative of the quantitative data shown in (**c**). **c** *Graph* showing the average number of fluorescent particles in each cell for control and PLCη2 KD groups. Five images were taken at random locations on the coverslip and nuclei and fluorescent particles which indicate PLCη2 interaction with LIMK-1 were counted according to a randomized counting method. **d**, **e** Fluorescent images [representative of quantitation in (**f**)] are taken from untreated and retinoic acid-treated Neuro2A cells, respectively (fluorescent particles corresponding to PLCη2-LIMK-1 interactions appear in *red*; DAPI-staining of nuclei in *blue*). **f** *Graph* showing the average number of fluorescent particles in untreated and retinoic acid-treated Neuro2A cells. Five micrographs were taken at random. Nuclei and fluorescent particles which indicate PLCη2 interaction with LIMK-1 were counted according to a randomized counting method. **g**, **h** Respective “close-up” images of the cells in (**d**, **e**) as indicated, where fluorescence is merged with *bright-field* view such that individual cells can be more easily visualized. *Error bars* represent standard error of the coefficient for five micrographs. The statistical significance (as determined by unpaired *t* test) is indicated as ****p < 0.0001. *Scale bars* correspond to 15 µm

LIMK-1 was previously identified as an interaction partner of PLCη2 using a bacterial hybrid screen (Popovics et al. 2013). In order to attempt to clarify the direct interaction of PLCη2 with LIMK-1, proximity ligand assays were performed using control and PLCη2 KD cells. In each of these cell lines, fluorescent particles, corresponding to interactions between PLCη2 and LIMK-1, were observed and were predominantly located within the cytosol, but some were also present in the cell nucleus (Fig. 3a–c). The number of fluorescent particles in the PLCη2 control cells was eightfold higher than in PLCη2 KD cells, indicating the staining to be specific. The assay was also performed with non-transfected Neuro2A cells grown for 4 days in the presence or absence of 20 μM retinoic acid. The retinoic acid-treated Neuro2A cells underwent normal differentiation, characterized by an enlargement of the cell body and neurite outgrowth (Fig. 3d–h). The number of fluorescent particles in retinoic acid-treated cells was approximately eightfold greater than the untreated cells. Particles were observed mainly in the cytoplasm and within growing neurites, as well as in the nucleus to a lower degree. These results confirm that PLCη2 and LIMK-1 do indeed interact and provide a strong indication that their interaction is important for retinoic acid-induced Neuro2A cell differentiation.

## Discussion

The Neuro2A cell is an established cell model for the examination of molecular events that regulate neuronal differentiation (Pankratova et al. 1994; Riboni et al. 1995; Carter et al. 2003; Kouchi et al. 2011), and upon stimulation with retinoic acid, they change from a stem cell-like morphology to a neuronal one, including formation and extension of neurites. An essential role for PLCη2 in neurite growth was previously demonstrated in Neuro2A cells (Popovics et al. 2014), which was further investigated in this study. Unlike most PLCs which comprise a non-calcium-sensing EF-hand-like domain (Bairoch and Cox 1990), PLCη2 has a functional EF-hand domain containing a canonical 12‐residue loop (EF‐loop 1) (Popovics et al. 2014). We reported that EF-loop 1 is responsible for activation of the enzyme by calcium through mutation of D256 (to Ala), a key calcium-binding residue in this domain, which resulted in an apparent ~tenfold reduction in calcium sensitivity in transfected COS-7 cells (Popovics et al. 2014). In an attempt to assess both whether PLCη2 activity is important for neurite growth and whether such involvement may be due to activation by calcium, we generated Neuro2A cells stably overexpressing wild-type PLCη2 and mutant forms of the enzyme (D256A and H460Q). Following retinoic acid treatment, there was a slight but significant increase in the percentage of differentiated cells observed in the wild-type PLCη2 stably transfected cells, relative to controls cells. Conversely, cells stably transfected with the H460Q mutant exhibited a dramatic reduction in the proportion of differentiated cells (~25-fold) relative to control cells after stimulation with retinoic acid. A comparable degree of differentiation was observed in the cells stably transfected with the D256A mutant relative to control after the treatment. These results reveal that expression of the H460Q mutant has a strong dominant-negative effect on neurite outgrowth. These results further highlight the essential role of PLCη2 in neurite outgrowth. In addition, they suggest that modulation of PLCη2 activity by calcium binding at the EF-hand domain may enhance, presumably through positive feedback of calcium signaling, but is not essential to retinoic acid-stimulated neurite growth. It is therefore likely that the principal mechanism by which PLCη2 is activated is not through calcium binding, but through another unknown mechanism. Recently, a new role has emerged for PLCη2 in secretion of cytoplasmic dense core vesicles in neuroendocrine cells. This is accomplished by PLCη2-mediated PtdIns(4,5)P_2_ hydrolysis following its activation by Ca^2+^. The decrease in PtdIns(4,5)P_2_ causes dysregulation of actin binding proteins which results in F-actin disassembly, thereby removing the physical barrier which would otherwise prevent dense core vesicle up-regulation to the plasma membrane (Yamaga et al. 2015). In this process, reorganization of the actin cytoskeleton is therefore dependent on Ca^2+^ activation of PLCη2. The authors did, however, show that this occurs only when intracellular Ca^2+^ levels are very high (~800 nM), and showed exocytosis of plasma membrane dense core vesicles at lower Ca^2+^ levels (~400 nM). This suggests that Ca^2+^ activation of PLCη2 may be involved in actin reorganization under certain conditions that significantly elevate intracellular Ca^2+^ levels.

To establish where in Neuro2A cells PLCη2 is specifically located, we examined the subcellular localization of endogenous PLCη2 by quantitative immuno-EM. This approach revealed the majority of endogenous PLCη2 to reside in the nucleus. We also observed a relatively high degree of PLCη2 to be present in the cytosol and to a lower extent, at the plasma membrane. The presence of PLCη2 in the nucleus and cytosol is consistent with previous immunofluorescent studies which also revealed a high degree of nuclear staining, corresponding to the presence of PLCη2, in these cells (Popovics et al. 2011). The PH domain of PLCη2 has been shown to bind phosphatidylinositol 3,4,5-trisphosphate (PtdIns(3,4,5)P_3_) with high affinity, a phospholipid which is abundant in Neuro2A cell nuclei (Popovics et al. 2011). Indeed PLCη2 and PtdIns(3,4,5)P_3_ were shown to co-localize in these cells to a large degree (Popovics et al. 2011), and hence this interaction could govern the cellular localization of PLCη2. If so, then it is also likely that PLCη2 resides in the nucleus of neuronal cells as they have also been reported to possess high levels of PtdIns(3,4,5)P_3_ in their nuclei (Neri et al. 1999; Kwon et al. 2010). The presence of other phospholipases such as PLCγ1, PLCδ4 and PLCβ1 in eukaryotic cell nuclei is well established (Martelli et al. 1992; Divecha et al. 1993; Matteucci et al. 1998; Ye et al. 2002; Liu et al. 1996). PLCβ1 is perhaps the best characterized of these and has proposed roles in mediating differentiation of C2C12 myoblasts (Faenza et al. 2003) and erythroleukemia cells (Fiume et al. 2005), mainly through regulation of gene expression. It is therefore possible that PLCη2 plays a similar role in neuronal differentiation. Evidence for a role in regulation of gene expression is supported by the observation that activation of retinoic acid response element (RARE)-associated gene expression is dramatically reduced in PLCη2 KD cells (Popovics et al. 2011). As with the cytoplasm, PLCη2 may also be responsible for regulating actin dynamics in the nucleus. Although β-actin has been identified in the nucleus, actin filaments have not been detected at interphase by common F-actin detecting methods (Sellers 2004). It has, however, been reported that ~20 % of nuclear actin forms polymeric structures, with rapid turnover (McDonald et al. 2006). Both cofilin and LIMK-1 possess nuclear localization sequences and are present in the nucleus (Abe et al. 1993; Goyal et al. 2006). More recently, it has emerged that cofilin is required for RNA polymerase II functioning and transcription elongation (Obrdlik and Percipalle 2011). It is therefore likely that PLCη2 is involved in regulating gene expression and actin-related processes in the nucleus; however, the precise role of nuclear PLCη2 remains to be established and must be probed further.

Another potential mechanism by which PLCη2 may contribute to neurite growth is through regulation of cytoskeletal dynamics. LIMK-1, a specific regulator of actin polymerization, was previously identified using a bacterial-2-hybrid screen as a putative interaction partner of PLCη2; however, a specific interaction of these two proteins within the cell was not shown (Popovics et al. 2011). It is well established that LIMK-1 deactivates cofilin family proteins by phosphorylation, which in turn prevents actin depolymerization and contributes to reorganization of the actin cytoskeleton (Ghosh et al. 2004; Lorenz et al. 2004). Accordingly, neurons from LIMK-1 knockout mice show reduced or deficient growth cones as well as abnormal dendritic morphology, synapse structures and spine development which manifest in behavioral changes such as the re-learning of spatial information (Meng et al. 2002). LIMK-1 also appears to have contradictory roles; activation of LIMK-1 by Semaphorin 3A (Aizawa et al. 2001), or fibrillar amyloid beta (Heredia et al. 2006) for example, leads to growth cone collapse and neurite deformation, respectively. Based on these studies, it appears that LIMK-1 is an essential protein for correct central nervous system development, but its regulation is important for normal physiology and alterations to its normal functioning leads to human diseases such as William’s syndrome, which is characterized by mild to moderate mental retardation (Meyer-Lindenberg et al. 2006).

To establish whether PLCη2 and LIMK-1 interact directly, we utilized a proximity ligand interaction assay which allowed quantification and localization of interactions between the two proteins. To attain a positive signal using this approach, proteins must be in the proximity of <30–40 nm; this distance is a good indication that proteins are close enough to one another to interact, or at the very least, participate in cross talk. By looking at compiled Z-stack images, we were able to determine that this putative interaction took place mainly in the cytosol and within growing neurites, suggestive of a role in regulating actin dynamics. Putative PLCη2-LIMK-1 interactions were also observed in the nucleus, albeit to a lower degree. Accordingly, LIMK-1 has a nuclear localization signal which drives translocation into the nucleus, and shuttling between the nucleus and the cytoplasm is indicated by presence of nuclear import and export sequences (Goyal et al. 2006). Upon cell differentiation, we observed a ~sixfold increase in fluorescent particles in Neuro2A cells, suggesting that these proteins interact to a higher degree during differentiation. This may in part be due the fact that PLCη2 protein expression is increased in Neuro2A cells following retinoic acid treatment (Popovics et al. 2013). Some evidence that PLCη2 activation occurs upstream from LIMK-1 comes from the observation that phosphorylation of LIMK-1 and CREB, a substrate of LIMK-1, is significantly reduced in retinoic acid-treated PLCη2 KD cells relative to control cells (Popovics et al. 2013). Also down-regulation of either of these genes significantly decreases neurite outgrowth in cultured cells (Endo et al. 2007; Popovics et al. 2013).

We previously proposed a model by which PLCη2 may regulate neuronal differentiation (Popovics et al. 2011), whereby the generation of secondary messengers, Ins(1,4,5)P_3_ and DAG impact upon cytoskeletal dynamics and gene expression, respectively. PLCη2 can be activated by Gβγ dimers, but as retinoic acid acts upon nuclear receptors rather than GPCRs, it would seem unlikely that the enzyme is activated this way in the current context. PLC isozymes such as PLCδ1 can be activated by kinases (Fujii et al. 2009), of which PLCη2 may also be a target. It is in theory possible that PLCη2 may be activated by LIMK-1; however, without knowledge of a specific activating phosphorylation site on PLCη2, this will be difficult to establish.

Upon PLCη2 activation, the level of PtdIns(4,5)P_2_ will likely decrease with subsequent elevations in Ins(1,4,5)P_3_ and DAG. Ins(1,4,5)P_3_ triggers calcium release from the intracellular stores to activate CaMKIV. The consequent stimulation of LIMK-1 activity leads to activation of CREB which initiates transcription of genes directing neuronal differentiation (Popovics et al. 2013). Here, we have clarified part of this model by demonstrating the direct association of PLCη2 and LIMK-1 in differentiating Neuro2A cells. This direct association could allow PLCη2 to directly activate LIMK-1 by promoting an active conformation upon association, or it is possible that this interaction simply allows PLCη2 to be “on hand” to modulate LIMK-1 activation via Ins(1,4,5)P_3_ release and CaMKIV activation. Changes in the nuclear DAG and PtdIns(4,5)P_2_ levels may also influence transcriptional activity through modulation of DAG-PKC or PtdIns(4,5)P_2_-chromatin interactions.

In conclusion, we reveal that PLCη2 activity in Neuro2A cells is important for retinoic acid-induced neurite growth but is not dependent upon calcium binding at EF-loop 1, highlighting the fact that activation of PLCη2 during this process does not primarily occur through calcium binding, but via another unknown mechanism. We also demonstrate that PLCη2 is present in the nucleus and cytosol of Neuro2A cells and reveal that the enzyme interacts directly with LIMK-1 in the cytosol and within growing neurites as well as inside the nucleus. This interaction has important implications for regulation of actin dynamics and expression of genes implicated in neuronal differentiation.
